# Comparative efficacy and safety of antibiotics used to treat acute bacterial skin and skin structure infections: Results of a network meta-analysis

**DOI:** 10.1371/journal.pone.0187792

**Published:** 2017-11-14

**Authors:** Julian F. Guest, Jaime Esteban, Anton G. Manganelli, Andrea Novelli, Giuliano Rizzardini, Miquel Serra

**Affiliations:** 1 Faculty of Life Sciences and Medicine, King’s College, London, United Kingdom; 2 Catalyst Health Economics Consultants, Rickmansworth, Hertfordshire, United Kingdom; 3 Department of Clinical Microbiology IIS-Fundacion Jimenez Diaz, UAM, Madrid, Spain; 4 Centre for Research in Health and Economics (CRES), University Pompeu Fabra, Barcelona, Spain; 5 Department of Health Sciences, University of Florence, Florence, Italy; 6 Department of Infectious Diseases, L. Sacco Hospital, Milan, Italy; 7 School of Clinical Medicine, Faculty of Health Science, University of the Witwatersrand, Johannesburg, SA; Kingston University, UNITED KINGDOM

## Abstract

**Objective:**

This NMA compared the efficacy and safety between IV antibiotics that are used in the current standard of care for managing adult patients (≥18 years of age) with ABSSSI.

**Methods:**

Comparators were chosen on the basis that both direct and indirect comparisons between the interventions of interest could be performed. Outcomes of the analysis were selected on the basis that they are frequently measured and reported in trials involving ABSSSI patients, and only published randomised control trials of any size and duration and with any blinding status were eligible for inclusion in the analysis. The NMA was performed using both a fixed-effect and random-effect model. Efficacy-related endpoints were (1) clinical treatment success and (2) microbiological success at TOC visit. Safety-related endpoints were (1) number of discontinuations due to AEs/SAEs, (2) patients experiencing AEs, (3) patients experiencing SAEs and (4) all-cause mortality.

**Results:**

Study interventions included daptomycin, dalbavancin, linezolid and tigecycline. Vancomycin was the comparator in all studies, except in two where it was linezolid and teicoplanin. The NMA showed that irrespective of patient subgroup, the likelihood of clinical and microbiological success with dalbavancin was statistically similar to the comparators studied. No statistically significant differences were observed between dalbavancin and any of the comparators in the discontinuation rate due to AEs/SAEs. In contrast, dalbavancin was associated with a significantly lower likelihood of experiencing an AE than linezolid, a significantly lower likelihood of experiencing a SAE than vancomycin and daptomycin, and a significantly lower risk of all-cause mortality than vancomycin, linezolid and tigecycline.

**Conclusion:**

Dalbavancin affords a promising, new alternative IV antimicrobial agent which is as effective as traditional therapies, but with the added benefit of enabling clinicians to treat patients with ABSSSI in different organisational settings. Notwithstanding, any introduction of an effective treatment with a differential mode of administration into healthcare systems must be followed by a change in clinical practice and patient management in order to fully achieve desirable economic outcomes.

## Introduction

The incidence of SSSI over a three year period (2009 to 2011) has been estimated at 496 per 10,000 person-years in the US [1]. The incidence is rising globally [2], thereby adding to an ever increasing health economic burden. The FDA defines ABSSSI as bacterial infections of the skin with a lesion size of at least 75 cm^2^ (measured by the area of redness, oedema, or induration) and include cellulitis, wound infection, and abscesses with surrounding cellulitis [3]. ABSSSI are caused by many different bacteria, most commonly Gram-positive bacteria including MRSA and MSSA [4, 5]. Numerous treatment options for ABSSSI are available including daptomycin, linezolid, tigecycline, teicoplanin and vancomycin, but there is limited evidence on their comparative effectiveness [6, 7], and treatment is becoming more difficult due to increasing antibiotic resistance [8–10].

Pan-European guidelines on the management of ABSSSI had not been published at the time of performing this analysis. Current Infectious Diseases Society of America guidelines [11] provide therapeutic recommendations for 46 purulent and non-purulent ABSSSI that are primarily based on the β-lactam class of antibiotics. When MRSA is a suspected pathogen the guidelines recommend empiric therapy with trimethoprim/sulfamethoxazole and doxycycline for purulent cases of moderate severity, and other agents, such as vancomycin, linezolid, or daptomycin, for treating severe purulent cellulitis. Irrespective of antimicrobial therapy, the guidelines recommend adequate source control for infection, such as draining of abscesses and debridement of nonviable tissues.

The majority of published RCTs have been compared to vancomycin and have not achieved statistically significant differences [6], although some studies favour linezolid over vancomycin [12–15]. Numerous meta-analyses have been conducted comparing the efficacy of vancomycin, linezolid, and other anti-microbial agents, and find clinical superiority for linezolid [5, 16–27]. However, most of these were only direct comparisons of two treatments and neglected the impact of indirect evidence.

Bayesian NMA is a statistical method which enables a direct and indirect comparison of treatments from multiple studies [28–30]. Two Bayesian NMAs have been conducted in ABSSSI caused by MRSA [5, 16]. One found higher success rates for linezolid, dalbavancin, and telavancin compared with daptomycin, vancomycin, tigecycline, but the analysis only included studies published up to 2010 [5]. The second was conducted in 2012 and compared vancomycin, linezolid, and ceftaroline [16]. A more recent NMA [31] suggested equivalence in clinical efficacy between vancomycin, daptomycin, linezolid, and novel antimicrobial agents in treating ABSSSI at TOC. However, the analysis reported uncertainty indicative of heterogeneity in the available evidence.

Recently approved antibiotics for the treatment of ABSSSI, such as dalbavancin [32, 33] further highlight the need for new comparative effectiveness research. Dalbavancin is a second-generation, semi-synthetic lipoglycopeptide antibiotic indicated for the treatment of ABSSSI in adults [32, 33]. This antibiotic has shown significant efficacy in the treatment of clinical infections caused by Gram-positive bacteria, including MRSA. Unlike other antibiotics, dalbavancin’s long half-life allows it to be administered as one dose or as a once-weekly rather than a daily dosing regimen [34], thereby decreasing the potential for patient non-adherence.

Additional antimicrobial prescribing options for cSSTIs are important for combating resistance to existing therapies and to improve patient outcomes. However, at the time of performing this study there were no direct comparative studies of dalbavancin with daptomycin, teicoplanin, vancomycin and linezolid. Accordingly, a systematic literature review [35, 36] comprising a network meta-analysis was conducted with the aim of comparing the relative efficacy and safety of dalbavancin with that of other IV antibiotics that are used in the current standard of care for managing patients with ABSSSIs.

## Methods

### Systematic literature review

A systematic literature review was conducted to identify the current available evidence in terms of clinical efficacy and safety of antibiotics used to treat ABSSSI. In the absence of direct head-to-head comparisons of treatments of interest, a NMA of dalbavancin, daptomycin, linezolid, teicoplanin, tigecycline and vancomycin was performed. The systematic review and NMA were conducted according to the principles set out in the Centre for Research and Dissemination (CRD) [37] and International Society for Pharmacoeconomics and Outcomes Research (ISPOR) guidelines [38, 39].

### Search strategy

The search strategy was based on a predefined PICOS [40] strategy (Table 1). The study population was defined as adult patients (≥ 18 years of age) of any gender with complicated ABSSSI (suspected or confirmed to be caused by Gram-positive bacteria) and suspected or confirmed MRSA treated in hospital.

**Table 1:** PICOS strategy for clinical evidence of dalbavancin in ABSSSI.

PICOS	Clinical Review
**Population**	Adult and paediatric patients of any gender with complicated ABSSSI (suspected or confirmed to be caused by Gram-positive bacteria)
**Intervention**	Studies of any intervention that has been or is currently used in the management of ABSSSIs will be considered eligible. The list of interventions included: ceftaroline fosamil, clindamycin, dalbavancin, daptomycin, linezolid, oritavancin, tedizolid, telavancin, tigecycline, tetracycline (minocycline or doxycycline), trimethoprim-sulfamethoxazole, vancomycin
**Comparator**	The same as the interventions
**Outcome**	• Clinical cure (resolution of symptoms and signs) • Microbiological cure (eradication of bacteria on wound culture), • Adverse events • Mortality (ABSSSI-related or all-cause)
**Study design**	Randomised controlled trials of any size and duration

Abbreviations: ABSSSI: acute bacterial skin and skin structure infection.

The following common interventions used for the treatment of complicated ABSSSI in Europe were included in the analysis:

■ Dalbavancin.
■ Daptomycin.
■ Linezolid.
■ Tigecycline.
■ Teicoplanin.
■ Vancomycin.

The comparators were chosen on the basis that both direct and indirect comparisons between the interventions of interest could be performed, and comprised any of the listed interventions with or without concomitant Gram-negative antibiotic administration.

The selected outcomes of the analysis were chosen on the basis that they are frequently measured and reported in trials involving ABSSSI patients. The outcomes of interest were:

■ Clinical treatment success.
■ Microbiological success.
■ Discontinuation due to AEs/SAEs.
■ Any AEs.
■ Any SAEs.
■ All-cause mortality.

Randomised controlled trials are the gold standard of clinical evidence. Hence, only published randomised control trials of any size and duration and with any blinding status were eligible for inclusion in the analysis.

Studies were excluded from the analysis if they were non-randomised or observational studies; review articles; lacked specific definitions and/or outcomes for SSTIs; contained pooled analyses or sub-group analyses of other studies; or lacked the interventions of interest.

The search strings used to interrogate PubMed and the Cochrane database without any language or time restrictions are summarised in S1 and S2 Tables. The searches were conducted in November 2014. Additionally, the ClinicalTrials.Gov database (https://clinicaltrials.gov) was searched in January 2015. Websites for the European Society of Clinical Microbiology and Infectious Disease, Interscience Conference on Antimicrobial Agents and Chemotherapy, IDWeek and International Society for Pharmacoeconomics and Outcomes Research were also searched.

The search terms were “skin structure infection” as conditions and “Ceftaroline OR clindamycin OR daptomycin OR linezolid OR oritavancin OR tedizolid OR telavancin OR tigecycline OR tetracycline OR trimethoprim OR vancomycin” as interventions. Additional methods to identify ongoing and recently completed research included searching the websites of health technology assessment and related agencies, professional associations and the key conferences to identify conference abstracts from the last 3 years.

### Network meta-analysis

The NMA was performed using WinBUGS version 1.4.3 [41], which employs Bayesian methods principles. Both a fixed-effect and random-effect model were developed and compared using the DIC [42].

A fixed-effect model assumes that all studies in the analysis are functionally identical and requires individual studies to use subjects that are as similar as possible. The aim of such an analysis is to compute a common effect size for the identified population and not to generalise to other populations and any variability in the effect size across individual studies is due to the fact that they simply employ different subjects from the same population.

A random-effect model can be used when all the studies in the analysis have been conducted independently and are unlikely to be functionally equivalent. Typically, the subjects or interventions in these studies differ in ways that would have impacted on the results, and therefore a common effect size should not be assumed. Accordingly, a random-effect model estimates a distribution of effect sizes (while the fixed-effect model estimates an individual effect size). This implies that the error term includes the variation across studies, which is more encompassing than the traditional random error term and the within-study variability. This also means that a random-effect analysis is able to generalise results to a range of scenarios.

The DIC is a measure of goodness of fit with complexity. The model with the smallest DIC is estimated to be the model that would best predict a replicate dataset which has the same structure as that currently observed and the residual deviance is a quality-of-fit statistic for the FE and RE models. It has been suggested that differences in DIC over 5 are important, while a difference of <3 is negligible [29]. The choice of whether to use a fixed or random effect model was based on the evaluation of the DIC statistic and heterogeneity assessment.

Statistical heterogeneity was assessed for each endpoint. Heterogeneity was defined as variability among studies such that it could influence the observed intervention effects, making them differ from each other more than would be expected due to random error alone.

For each pairwise comparison that was informed by at least two trials, an ordinary meta-analysis was performed (using the standard frequentist approach to pairwise meta-analysis) [43]. Heterogeneity was assessed using (1) the I-squared (I^2^) statistic, which describes the percentage of variation across studies that is due to heterogeneity rather than chance and (2) the tau-squared value (τ2) which measures an estimate of the between-study variance in a random-effects model and (3) the p-value of the heterogeneity statistic Q (which is the weighted sum of squared differences between individual study effects and the pooled effect across studies, with the weights being those used in the pooling method) [43]. I^2^ values of 0–40%, 30–60%, 50–90% and 75–100% were defined as ‘not important’, ‘moderate’, ‘substantial’ and ‘considerable’ heterogeneity respectively [43].

In addition to statistical heterogeneity, an explorative analysis was carried out in order to list all the potential sources of heterogeneity. The potential heterogeneity sources were partitioned into two distinct classes:

■ *Clinical heterogeneity* refers to the potential sources originating from differences in patient characteristics that could influence study-specific effect estimates. Such differences can be due to varying inclusion and exclusion criteria between the trials or to peculiar characteristics of the sampled individuals.
■ *Methodological heterogeneity* includes potential causes of bias explained by study design and variation in the definition of the outcomes of interest.

Inconsistency (i.e. differences between direct and indirect estimates) was assessed by the Bucher method [30]. For a particular treatment comparison, with estimates based on both direct and indirect evidence, the statistical significance (two sided p-value) of the difference between the direct and indirect evidence was tested.

### Endpoints of the network meta-analysis

All endpoints included in the NMA were dichotomous and were summarised as an OR. The efficacy-related endpoints were (1) clinical treatment success at TOC visit and (2) microbiological success at TOC visit. The safety-related endpoints were (1) number of discontinuations due to AEs/SAEs, (2) patients experiencing AEs, (3) patients experiencing SAEs and (4) all-cause mortality.

The clinical treatment success and microbiological success considered in the NMA were those reported at the TOC visit in those studies where this visit took place. In the event that the clinical or microbiological success was not assessed/reported at TOC, the assessment at the EOT visit was used instead.

Two separate analyses were conducted for each outcome: (1) one for studies including adults only and (2) one which also included those studies with a mixed population of children and adults.

## Results

### Study selection

**Fig 1:**
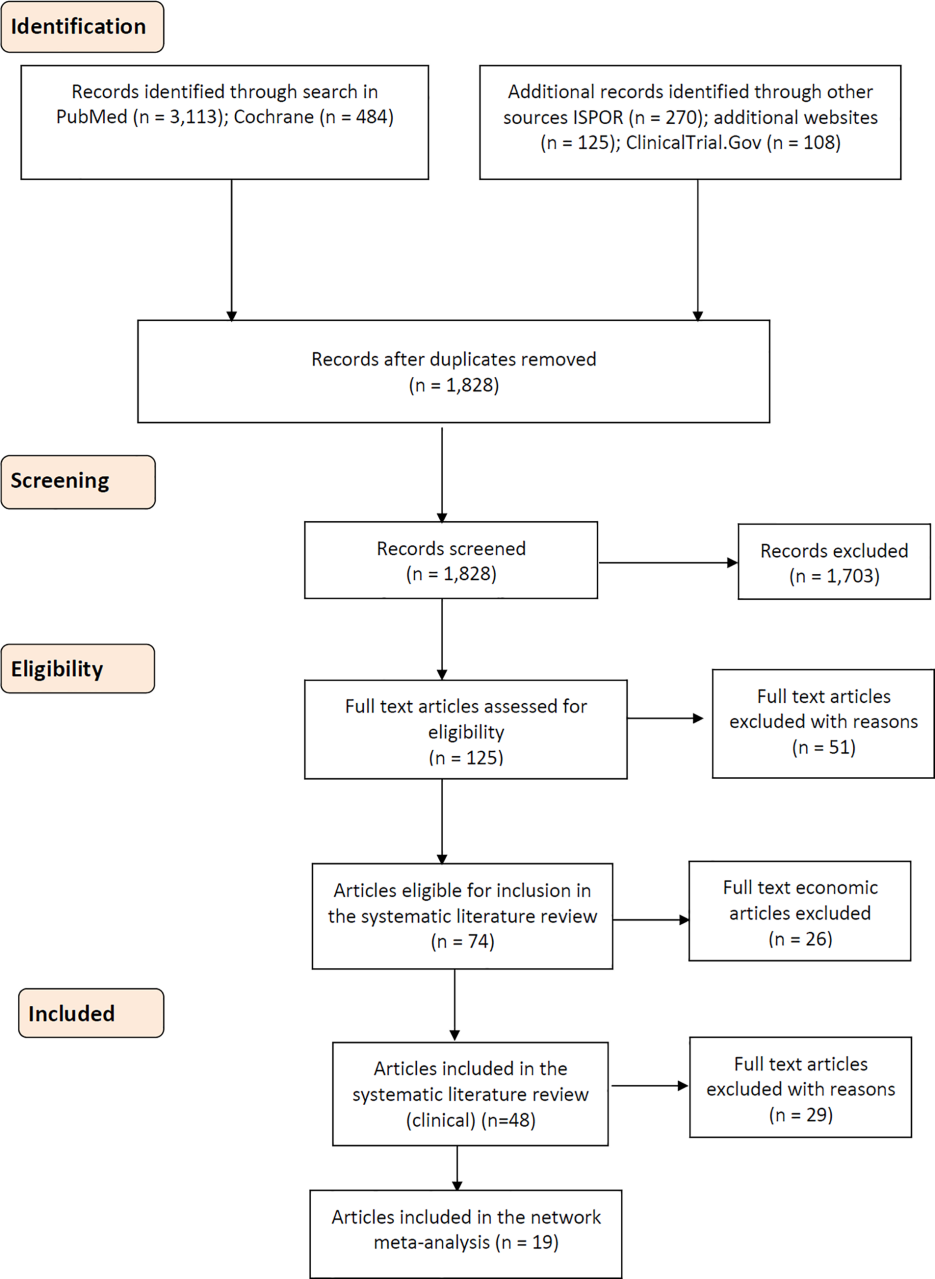
PRISMA flow diagram with the clinical studies identified through the predefined search strategy.

**Fig 2:**
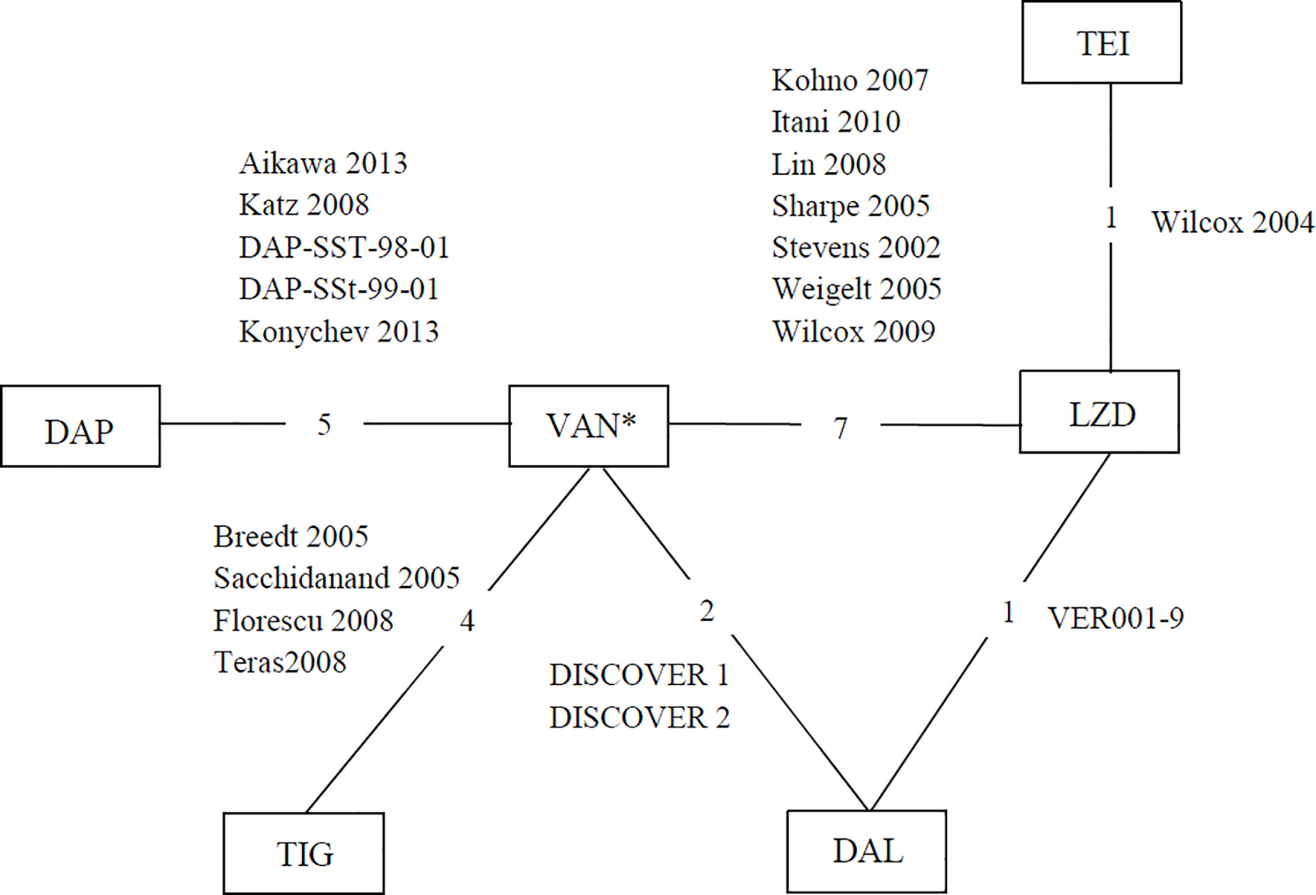
Network of the studies used in the meta-analysis. Abbreviations: DAL: dalbavancin; DAP: daptomycin; LZD: linezolid; TEI: teicoplanin; VAN: vancomycin; TIG: tigecycline. *Includes studies where the comparator was vancomycin, vancomycin plus aztreonam. Or vancomycin plus conventional treatment. Each node represents a treatment and the connecting lines indicate pairs of treatments that have been directly compared in one RCT. The numbers in the connecting lines indicate the number of relevant studies for each treatment comparison.

The systematic literature review yielded 48 relevant references of which 46 distinct studies provided clinical and safety-related data (Fig 1). Of these, 17 studies reported in 19 references met the inclusion criteria for the NMA. A summary of these studies is presented in Tables 2 and 3. The 29 clinical studies that were excluded are listed in S3 Table. The network connecting all the studies included in the different analyses is illustrated in Fig 2.

**Table 2:** Summary information of all RCTs included in the network meta-analysis.

Trial acronym	Source	Design	Population	Treatment (sample size)	Primary Endpoint
Aikawa 2013	[49]	R/MC/Ph3/OL	Confirmed MRSA-cSSTI	DAP (n = 88) VAN (n = 22)	Clinical and microbiological response at TOC
DAP-SST-98-01 and DAP-SST-99-01	[45]	R/MC/Ph3/SB	cSSSI	DAP (n = 534) PRP (cloxacillin, nafcillin, OXA, or flucloxacillin), or VAN (n = 558)	Clinical response at TOC
DISCOVER 1	[32]	R/MC/Ph3/DB	ABSSSI	DAL (n = 288) VAN, with an option to switch to oral LZD (n = 285)	Early clinical response (after 48 to 72 hours of therapy)
DISCOVER 2	[32]	R/MC/Ph3/DB	ABSSSI	DAL (n = 371) VAN, with an option to switch to oral LZD (n = 368)	Early clinical response (after 48 to 72 hours of therapy)
Breedt 2005	[44]	R/MC/Ph3/DB	cSSSI	TIG (n = 275) VAN + AZA (n = 271)	Clinical response at TOC
Florescu 2008	[53]	R/MC/Ph3/DB	Confirmed MRSA-cSSSI, cIAI or pneumonia	TIG (n = 118; patients with cSSSI: 83) VAN or LZD (n = 39; patients with cSSSI: 27)	Clinical response at TOC
Itani 2010	[50]	R/MC/Ph4/OL	Confirmed MRSA-cSSTI	LZD (n = 537) VAN (n = 515)	Clinical cure at EOT
VER001-9	[33]	R/MC/Ph3/DB	cSSSI	DAL (n = 571) LZD (n = 283)	Clinical success at TOC
Katz 2008	[46]	R/MC/SB	cSSSI	DAP (n = 48) VAN (n = 48)	Clinical response at 7–14 days post-therapy
Kohno 2007	[12]	R/MC/OL	Confirmed MRSA-cSSTI, pneumonia or sepsis	LZD (n = 100; patients with cSSTI: 31) VAN (n = 51; patients with cSSTI:17)	Clinical and microbiological outcome EOT
Konychev 2013	[54]	R/MC/Ph3/OL	cSSTI with or without bacteraemia	DAP (n = 81) SSP or VAN (n = 39)	Clinical success at TOC
Lin 2008	[51]	R/MC/Ph3/DB	cSSTI or pneumonia	LZD (n = 71; patients with cSSTI: 33) VAN (n = 71; patients with cSSTI: 29)	Effective treatment rate at EOT and FU
Sacchidanand 2005	[47]	R/MC/Ph3/DB	cSSSI	TIG (n = 295) VAN + AZA (n = 298)	Clinical cure rate at TOC
Sharpe 2005	[13]	R/OL	Confirmed MRSA-cSSTI	LZD (n = 30) VAN (n = 30)	Clinical and microbiological outcomes
Stevens 2002	[14]	R/MC/OL	Presumed or confirmed MRSA-cSSTI, pneumonia or urinary tract infection	LZD (n = 240; patients with cSSTI: 102) VAN (n = 220; patients with cSSTI: 108)	Clinical and microbiological outcomes at TOC
Teras 2008	[48]	R/MC/Ph3/DB	cSSSI	TIG (n = 196) VAN + AZA (n = 191)	Clinical response at TOC
Weigelt 2005	[15]	R/MC/OL	Presumed or confirmed MRSA-cSSTI	LZD (n = 476) VAN (n = 454)	Clinical response at TOC
Wilcox 2004	[52]	R/MC/Ph3/OL	cSSTI, pneumonia, right-sided endocarditis, or bacteraemia	LZD (n = 215; patients with cSSTI: 123) TEI (n = 215; patients with cSSTI: 117)	Clinical outcome at the EOT and TOC visits
Wilcox 2009	[55]	R/MC/OL	Presumed or confirmed MRSA-cSSSI or CRBSI	**LZD** (n = 363; patients with cSSSI: 164) **VAN** (n = 363; patients with cSSSI: 151)	Microbiologic outcome at TOC

Abbreviations: ABSSSI: acute bacterial skin and skin structure infection; AZA: aztreonam; CRBI: catheter-related bloodstream infection; cSSSI: complicated skin and skin structure infection; cSSTI: complicated skin and soft tissue infection; DAL: dalbavancin; DAP: daptomycin; DB: double blinded; EOT: end of treatment; ITT: intent to treat; LZD: linezolid; MC: multicentre; MRSA: methicillin-resistant Staphylococcus aureus; OL: open label; OMA: omadacycline; OXA: oxacillin; Ph: phase; R: randomised; SB: single blinded; SSP: semi-synthetic penicillin; TEI: teicoplanin; TIG: tigecycline; TOC: test of cure; tx: treatment; TZD: tedizolid phosphate; VAN: vancomycin.

**Table 3:** Clinical trials included in the network meta-analysis.

	Treatments included						NMA Analysis								
							Analysis group		Outcomes						
Trial acronym	VAN	LZD	DAL	DAP	TEI	TIG	Adults only	Adults and mixed population	Clinical success	Microbiological success		Discontin-uation due to AEs/SAEs	Rate of AEs	Rate of SAEs	All-cause mortality
Aikawa 2013 [49]	Y			Y			Y	Y	Y	Y	Y		Y	Y	Y
DAP-SST-98-01* and DAP-SST-99-01* [45]	Y			Y			Y	Y	Y		Y		Y	Y	Y
Breedt 2005 [44]	Y					Y	Y	Y	Y	Y	Y		Y	Y	Y
DISCOVER 1 [32]	Y		Y				Y	Y	Y	Y	Y		Y	Y	Y
DISCOVER 2 [32]	Y		Y				Y	Y	Y	Y	Y		Y	Y	Y
Florescu 2008 [53]	Y					Y	Y	Y	Y						
Itani 2010 [50]	Y	Y					Y	Y	Y	Y			Y		Y
Katz 2008 [46]	Y			Y			Y	Y	Y	Y	Y		Y	Y	Y
Kohno 2007 [12]	Y	Y					Y	Y	Y	Y					
Konychev 2013 [54]	Y			Y			Y	Y	Y	Y	Y		Y	Y	Y
Lin 2008 [51]	Y	Y					Y	Y	Y	Y					
Sacchidanand 2005 [47]	Y					Y	Y	Y	Y	Y	Y		Y		Y
Sharpe 2005 [13]	Y	Y					Y	Y	Y						
Stevens 2002 [14]**	Y	Y						Y	Y						
Teras 2008 [48]	Y					Y	Y	Y	Y	Y	Y			Y	Y
VER001-9 [33]		Y	Y				Y	Y	Y	Y	Y		Y	Y	Y
Weigelt 2005 [15]	Y	Y					Y	Y	Y		Y		Y	Y	Y
Wilcox 2004 [52]**		Y			Y			Y	Y						
Wilcox 2009 [55]**	Y	Y						Y	Y	Y					

*Pooled analysis of these two studies was reported in Arbeit et al [45, 49].

**These studies were included only in the network for the mixed population analysis.

Abbreviations: DAL: dalbavancin; DAP: daptomycin; LZD: linezolid; TEI: ticoplanin; TIG: tigecycline; VAN: vancomycin.

### Population characteristics

All studies included in the NMA recruited patients with cSSSI. However, the definition of cSSSI differed across studies. In two studies [32] patients had to have an ABSSSI which was defined as an infection with a minimum lesion size of 75 cm^2^, either involving deeper soft tissue or requiring significant surgical intervention. The included infections were major cutaneous abscesses, surgical site or traumatic wound infection, and cellulitis. In addition to the presence of erythema, all patients were required to have at least two of the following signs: purulent drainage/discharge, fluctuance, heat/localized warmth, tenderness to palpation or swelling/induration. Furthermore, the infection severity had to be such that a minimum of 3 days of IV therapy was considered appropriate for managing the infection.

The study population was defined as adults with cSSSI in six of the studies [33, 44–48]. In a further four studies [13, 15, 49, 50] the study population consisted of adults with cSSSI, but MRSA had to be isolated from specimens before patients were recruited. In another three studies [14, 51, 52] the study indication included pneumonia and cSSTI caused by Gram-positive bacteria (including MRSA) and in two more studies [12, 53], the study population had pneumonia and bacteraemia as well as cSSSI. However, while in one study [12] only patients with MRSA-related serious infections could be included, the other [53] included patients infected with vancomycin-resistant *E*. *faecium* or *E*. *faecalis* or MRSA, either isolated alone or as part of a polymicrobial infection. In addition, in another study [54] patients with cSSTIs with or without bacteraemia were included. In contrast, bacteraemia was an exclusion criterion in two other studies [45, 49]. Notwithstanding the above, the study population in one study [55] differed from all the other studies since patients with central venous, pulmonary artery, or arterial catheter in place for 13 days and suspected catheter-related infection were eligible for participation in the study as were patients with *S*. *aureus* bacteraemia who were to undergo echocardiography to rule out endocarditis. However, the primary analysis population was restricted to patients with catheter-related blood infections and cSSSI. Hence, the primary disease differed across studies, although the clinical, microbiological and safety-related outcomes included in the NMA related only to the outcomes for cSSSI.

The study population of the different NMA studies also differed in terms of patients’ age. The majority of studies recruited patients aged ≥18 years. However, one study [49] recruited patients aged ≥20 years, another [54] recruited those aged ≥65 years and three others [14, 52, 55] recruited patients aged ≥13 years. The last three studies [14, 52, 55] comprised the mixed population analyses.

### Interventions

The study interventions in the NMA included daptomycin (5 studies), dalbavancin (3 studies), linezolid (8 studies) and tigecycline (4 studies). The dosage of an intervention tended to be the same across studies, but the dosage and treatment duration differed depending on the nature of the infection.

In all studies where daptomycin was the study intervention, it was administered at a dose of 4 mg/kg IV once-daily if patients did not have bacteraemia [45, 49] and at 6 mg⁄kg four times a day for 14–42 days if bacteraemia was present [46, 54]. In two studies the treatment duration was 5–14 [46] and 7–14 [54] days if no *S*. *aureus* bacteraemia was present and for 10–28 days and 14–42 days, respectively if bacteraemia was present.

The dose of dalbavancin was the same in all three studies where it was administered as one 1000 mg IV dose on day 1, followed by a 500 mg dose on day 8. Similarly, the dosage of tigecycline was the same in all studies, having been administered as an initial IV dose of 100 mg followed by 50 mg twice a day.

Linezolid was administered as 600 mg IV every 12 hours in all studies. However, in four studies IV linezolid could be switched to the oral formulation at the investigator’s discretion [12, 15, 50, 52]. The minimum duration of treatment differed depending on the infection type. In general, the minimum duration was 7 days for patients with cSSTI [51] or urinary tract infection [14], 10 days for patients with pneumonia [14, 51], and 14 days for patients with bacteraemia [14].

### Comparators

Vancomycin was the comparator in all studies included in the NMA, except in two where the comparator was linezolid [33] and teicoplanin [52].

IV vancomycin 1 g every 12 hours was the comparator in five studies [12–15, 55]. In another study [51], 1 g was administered if the patient was aged ≤60 years, but the dose was reduced to 0.75 g in patents aged >60 years. The same dose was administered in all the studies irrespective of the infection type. The minimum treatment duration was 7 days for patients with cSSTI [51] or urinary tract infection [14], 10 days for patients with pneumonia [14, 51], and 14 days for patients with bacteraemia [14].

In four studies [45, 49, 54] the comparator was either vancomycin (if MRSA or penicillin-allergy) or a penicillinase-resistant penicillin. The dosage of vancomycin was 1 g every 12 hours in all the studies. However, in one study [54] the dose of the penicillin differed between patients with bacteraemia (2 g every 4 hours) and without (2 g every 6 hours). In this study, the treatment duration also differed between patients with (10–28 days) and without (5–14 days) bacteraemia. In another study [53], the comparator was IV vancomycin if patients had MRSA infection, but linezolid if they had a VRE infection.

In two studies [32], the comparator was IV vancomycin, but patients could switch to oral linezolid after 3 days of therapy if:

- In the previous 24 hours, a patient had 4 temperature measurements, each separated by approximately 6 hours, in which all 4 measurements were ≤37.6°C/99.7°F.
- There was unequivocal improvement in some or all of the clinical signs of the ABSSSI under study; if some signs had not improved, none should have worsened.

Aztreonam and other drugs with Gram-negative coverage were permitted adjunctive therapies in most studies. However, it was used in combination with vancomycin in three studies [44, 47, 48], while vancomycin was administered as 1 g IV over 60 min, followed by 2 g aztreonam over 60 min, twice a day. In another study, [44] aztreonam could be discontinued after 48 h, according to the investigator’s clinical judgment.

Linezolid was the comparator in one study [33] in which patients were randomised to receive linezolid 600 mg intravenously or intravenously/orally every 12 h for 14 days. In another study [52] patients received IV or intramuscular teicoplanin as the comparator for up to 28 days.

### Study characteristics

All studies were randomised and included an active control. However, there were several differences in study design. Eight studies [33, 44, 47, 48, 51, 53, 56, 57] were all double-blinded, two were single blinded [12–15, 45, 46, 49, 50, 52, 54, 55] and other studies were all open label [12–15, 45, 46, 49, 50, 52, 54, 55]. In addition, all studies were phase III except for one study [50] which was phase IV; the phase was not stated in another [13].

Five studies were conducted in a single country, of which one was conducted in China [51], two in Japan [12, 49] and two in the US [13, 46]. The other studies were multinational and included several countries and several continents. In this respect, one study [48] reported the outcomes for the European cohort of a larger study. The results of the full study population do not seem to have been published. The systematic literature review [35, 36] conducted prior to the NMA did not identify any additional publications associated with this study.

The timing of the studies also varied, and ranged between 1998 and 2012 for those studies which provided information on the timing of the study. However, the timing is not mentioned in three studies [13, 47, 54].

In all studies, the outcomes were based on clinical and microbiologic assessments. The endpoints assessed were in line with those recommended by the FDA and included the clinical and microbiological success. However, the primary endpoint differed across studies. Similarly, the timing of assessment and the analysis population were not consistent across studies.

### Primary endpoint

The primary endpoint was clinical success at TOC in three studies [33, 45, 54]. However, the TOC visit took place between 12 and 16 days after completion of treatment at day 14 in one of these studies [33] and 7–14 days post-treatment in another [54]. The definition of clinical success was the same in all three studies. Clinical success was defined as improvement in signs and symptoms of SSSI such that no further antibacterial therapy was required.

In one study [49], the primary endpoint was defined as the clinical and microbiological response at TOC which took place 7–14 days of the last dose of study medication. Clinical and microbiological responses were confirmed by the Efficacy Adjudication Committee. In another study [12] the primary endpoint was also the clinical and microbiological response, but at the EOT and at the follow-up evaluation at 5–16 days post-treatment. While in another study [55] the primary end point was microbiologic outcome at TOC which took place 7 to 14 days after treatment. Clinical success at TOC was a secondary endpoint.

In all the other studies the primary endpoint was defined as the clinical outcome or response. The timing of assessment of the clinical outcome was TOC in most studies, but the timing of the TOC visit differed across studies. The TOC visit took place at different times:

- between 12 and 92 days after the last dose [44, 47, 48].
- between 12 and 37 days after the last dose of the study drug was administered [53].
- between 7 and 14 days after the last dose of the study drug was administered [46].
- between 7 and 28 days after the last dose of the study drug was administered [51].
- between 15–21 days after the end of therapy [14, 52].
- 10 days after the end of treatment [13].
- 7 days after the end of treatment [15].

In two studies [51, 52] the primary endpoint included the clinical outcome at the EOT visit which took place within 72 h after the last dose of study medication. In two studies [32], the primary outcome measure was clinical response at 48 to 72 hours after study drug initiation, as defined programmatically based on measurements of ABSSSI lesion size and temperature. However, the end of treatment endpoint was noted to be of special interest to regulators from the European Union. In another study [13] the primary endpoint is not specified, but both clinical and microbiological responses are assessed at TOC which took place 10 days after end of treatment.

### Network meta-analysis

All the aforementioned studies included an ITT population which was defined either as all patients who met the selection criteria or were randomised [44, 47, 48, 53, 54], or as all randomised patients who received at least one dose of study medication [12, 14, 15, 32, 33, 45, 46, 49–52, 55]. The NMA was restricted to the ITT populations which was defined as all randomised patients in each group.

The outcome at TOC was considered to be the primary clinical response. When this was not available the outcome at EOT was used in the NMA. Accordingly, it should be noted that for two studies [32], no TOC visit was pre-planned. However, the study protocols planned a visit at Day 28 (between Day 26 and 30) from randomisation, which was considered as being equivalent to the TOC visit for the NMA.

A summary of the model fit statistics for both the FE and RE models for each of the outcomes and patient sub-groups analysed is provided in Table 4.

**Table 4:** Model fit statistics for all endpoints and patient subgroups.

Endpoint	Patient subgroup	Model	Residual deviance	DIC	pD
Clinical success	Adults	FE	36.38	214.61	20.04
		RE*	33.15	215.23	24.10
	Mixed	FE	41.81	252.00	24.03
		RE*	39.91	253.37	27.50
Microbiological success	Adults	FE	44.85	171.31	17.04
		RE*	26.14	159.46	23.87
	Mixed	FE	46.99	183.44	18.03
		RE*	29.10	173.05	25.51
Discontinuation due to AEs	Adults	FE*	22.25	117.58	14.78
		RE	20.31	118.26	17.45
Patients with AEs	Adults	FE*	22.95	162.99	15.05
		RE	21.52	164.15	17.73
Patients with SAEs	Adults	FE*	21.93	115.76	13.91
		RE	19.25	115.97	16.91
All-cause mortality	Adults	FE*	21.26	98.75	15.20
		RE	21.16	100.35	16.93

*Model which was deemed a more adequate fit for each analysis.

Abbreviations: DIC: Deviance Information Criterion; FE: fixed-effect; pD: leverage or number of parameters; RE: random effect.

The number of data points (one data point from each arm of each study) that each residual deviance should be compared with is:

- 32 for clinical success in the adults-only studies.
- 38 for clinical success in the mixed population studies.
- 26 for microbiological success in the adults-only studies.
- 28 for microbiological success in the mixed population studies.
- 22 for the discontinuation due to AEs/SAEs.
- 22 for rate of AEs.
- 20 for rate of SAEs.
- 24 for all-cause mortality.

Statistical heterogeneity (Table 5) was found to be considerable in clinical and microbiological success (adults) for dalbavancin versus vancomycin, substantial in microbiological success for linezolid (adults and mixed populations) and substantial for discontinuation due to AEs for tigecycline.

**Table 5:** Heterogeneity for the different pairwise meta-analyses used in the network meta-analysis.

Endpoint	Patient subgroup	Pairwise comparison	I^2^	Tau^2^	p-value
Clinical success	Adults	DAL vs VAN	76.8%	0.17	0.038
		DAP vs VAN	0%	0	0.424
		LZD vs VAN	52.5%	0.061	0.078
		TIG vs VAN	0%	0	0.964
	Mixed	LZD vs VAN	37.1%	0.032	0.146
Microbiological success	Adults	DAL vs VAN	85.6%	0.672	0.009
		DAP vs VAN	35.3%	0.221	0.213
		LZD vs VAN	63.9%	0.719	0.040
		TIG vs VAN	60.2%	0.221	0.081
	Mixed	LZD vs VAN	58.3%	0.300	0.048
Discontinuation due to AEs/SAEs	Adults	DAL vs VAN	0%	0	0.580
		DAP vs VAN	0%	0	0.660
		TIG vs VAN	61.7%	0.448	0.074
Patients with AEs	Adults	DAL vs VAN	0%	0	0.372
		DAP vs VAN	0%	0	0.516
		TIG vs VAN	0%	0	0.865
Patients with SAEs	Adults	DAL vs VAN	17.3%	0.047	0.272
		DAP vs VAN	37.5%	0.214	0.187
		TIG vs VAN	0%	0	0.394
All-cause mortality	Adults	DAL vs VAN	0%	0	0.400
		DAP vs VAN	0%	0	0.432
		LZD vs VAN	0%	0	0.810
		TIG vs VAN	0%	0	0.856

Abbreviations: DAL: dalbavancin; DAP: daptomycin; LZD: linezolid; TEI: teicoplanin; VAN: vancomycin; TIG: tigecycline.

Based on the model fit statistics and the heterogeneity results, the RE model was considered to provide the most adequate fit for the efficacy endpoints, The FE model was the most adequate for the safety-related outcomes, due in part to its ability to assist in controlling for unobserved heterogeneity [38, 39], such as different age distributions.

The NMA results for each of the endpoints by population group for each comparator are shown in Table 6. Only the results for the best fit model are displayed (RE for efficacy outcomes and FE for safety), but similar results were observed for the other model.

**Table 6:** Results for the efficacy and safety-related endpoints from the network meta-analysis.

Endpoint	Patient subgroup	Model (RE/FE)	Pairwise comparison	OR (CrI)
Clinical Treatment Success	Adult	RE	DAL vs VAN	0.99 (0.68; 1.51)
			DAL vs LZD	0.69 (0.41; 1.00)
			DAL vs DAP	1.05 (0.61; 2.10)
			DAL vs TIG	1.18 (0.71; 2.10)
	Mixed Population	RE	DAL vs VAN	0.96 (0.69; 1.35)
			DAL vs LZD	0.73 (0.50; 1.02)
			DAL vs DAP	1.00 (0.63; 1.76)
			DAL vs TIG	1.14 (0.72; 1.83)
			DAL vs TEI	1.67 (0.44; 7.37)
Microbiological success	Adult	RE	DAL vs VAN	1.31 (0.40; 4.93)
			DAL vs LZD	0.53 (0.11; 1.85)
			DAL vs DAP	1.85 (0.32; 12.72)
			DAL vs TIG	2.29 (0.44; 14.36)
	Mixed Population	RE	DAL vs VAN	1.21 (0.41; 4.01)
			DAL vs LZD	0.61 (0.16; 1.87)
			DAL vs DAP	1.71 (0.33; 10.11)
			DAL vs TIG	2.10 (0.47; 11.32)
Discontinuation due to AEs/SAEs	Adult	FE	DAL vs VAN	1.08 (0.59; 1.98)
			DAL vs LZD	1.24 (0.68; 2.30)
			DAL vs DAP	1.28 (0.53; 3.09)
			DAL vs TIG	1.43 (0.65; 3.23)
Patients experiencing AEs	Adult	FE	DAL vs VAN	0.85 (0.70; 1.03)
			DAL vs LZD	**0.78 (0.62; 0.98)**
			DAL vs DAP	1.05 (0.76; 1.46)
			DAL vs TIG	0.78 (0.59; 1.02)
Patients experiencing SAEs	Adult	FE	DAL vs VAN	**0.54 (0.30; 0.96)**
			DAL vs LZD	0.99 (0.61; 1.66)
			DAL vs DAP	**0.48 (0.24; 0.95)**
			DAL vs TIG	0.58 (0.25; 1.35)
All-cause mortality	Adult	FE	DAL vs VAN	**0.26 (0.05; 0.93)**
			DAL vs LZD	**0.20 (0.04; 0.77)**
			DAL vs DAP	0.34 (0.05; 1.71)
			DAL vs TIG	**0.06 (0.00; 0.44)**

Abbreviations: AE: adverse event; CrI: credible interval; DAL: dalbavancin; DAP: daptomycin; FE: Fixed Effect; LZD: linezolid; RE: random effect; SAE: serious adverse event; TEI: teicoplanin; TIG: tigecycline; VAN: vancomycin.

In **bold**, statistically significant.

The NMA shows that irrespective of the patient subgroup analysed (adults-only or mixed adult and children population), the likelihood of clinical and microbiological success with dalbavancin was statistically similar to those with vancomycin, linezolid, daptomycin, tigecycline and teicoplanin.

**Fig 3:**
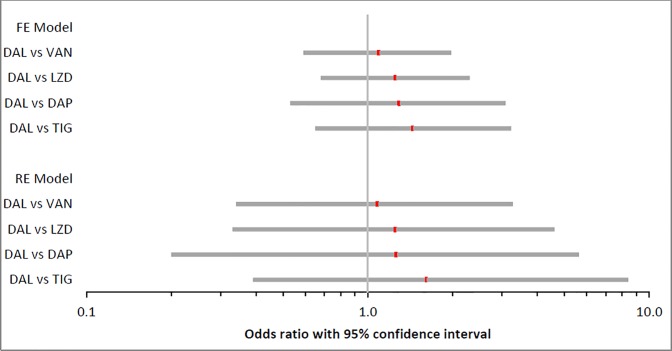
Forest plot on a log scale of the Odds ratios between dalbavancin and all other treatments for discontinuation due to AEs/SAEs for adults only (Odds ratio >1 favours DAL).

In terms of safety, no statistically significant differences were observed between dalbavancin and any of the comparators for the discontinuation rate due to AEs/SAEs (Fig 3). In contrast, dalbavancin was associated with a significantly lower likelihood of experiencing an AE than linezolid, a significantly lower likelihood of experiencing a SAE than vancomycin and daptomycin, and a significantly lower risk of all-cause mortality than vancomycin, linezolid and tigecycline. However, these significant findings should be viewed with caution given that populations and underlying diseases differed markedly across the studies.

The large credible intervals observed for some of the paired comparisons are due to the low number of studies included in some of the comparisons.

## Discussion

A NMA was conducted to compare the efficacy and safety of dalbavancin with that of IV comparators, since there were only direct comparative studies of dalbavancin with, vancomycin and linezolid. There were some differences in the methodology employed and patient populations between the identified trials. However, the studies were deemed sufficiently similar to derive reasonable estimates of the comparative efficacy and safety of the treatments. Despite different timings of assessment of clinical and microbiological outcomes and the different analysis sets defined in the different studies, all NMA analyses were restricted to the ITT population.

Some previous meta-analyses of the treatment of ABSSSIs found clinical superiority in favour of linezolid over vancomycin, with ORs ranging between 1.57, 1.40 and 1.67 [21, 25, 27]. A more recent meta-analysis [18] also found linezolid to be superior to vancomycin and other antibiotics with an OR of 1.61. However, these studies were restricted to direct comparisons and neglected indirect evidence. One previous NMA found both linezolid and daptomycin to be superior to vancomycin, dalbavancin, telavancin and tigecycline for MRSA complicated skin and soft tissue infections [5]. However, the search was conducted in 2008 and was limited to only 14 studies in MRSA.

Two further meta-analyses compared daptomycin with non-daptomycin treatments in SSSIs; one due only to MRSA [22] and another due to both MRSA and MSSA [17]. The MRSA-only study derived an OR of 0.89 for daptomycin relative to other treatments, whereas the MRSA and MSSA study derived an OR of 1.05 for daptomycin relative to vancomycin for clinical success. These findings were consistent with the estimates from a more recent NMA [31].

Even though most of the studies in this NMA have included mainly patients infected with MSSA and/or MRSA, other organisms have also been included in some studies, such as *Streptococcus pyogenes* [14] or even vancomycin-resistant enterococci [53]. Dalbavancin has shown good *in vitro* activity against most of these organisms (especially *S*. *aureus* [58]), and even other Gram-positive organisms, including coagulase-negative staphylococci, *Listeria*, *Corynebacterium*, *Micrococcus* or *Bacillus sp*. [59]. Moreover, in experimental models dalbavancin has been efficacious even in adverse situations such as foreign body infection [60] or against glycopeptide non-susceptible *S*. *aureus* strains [61]. These studies suggest that dalbavancin has sufficient *in vitro* data to support its use in the treatment of SSTI, where staphylococci and streptococci represent the main etiological agents in all series.

This present NMA generated relatively wide credible intervals surrounding some of the OR estimates. Despite this overall uncertainty, the analysis suggests that dalbavancin is not significantly different from the other agents with respect to efficacy as measured by clinical treatment success and microbiological success. In terms of safety, dalbavancin does not differ significantly from the current standard of care in terms of tolerability (i.e. discontinuation due to AEs/SAEs). In contrast, dalbavancin was associated with a significantly lower likelihood of experiencing an AE than linezolid, a significantly lower likelihood of experiencing a SAE than vancomycin and daptomycin, and a significantly lower risk of all-cause mortality than vancomycin, linezolid and tigecycline. However, the significant advantage for dalbavancin should be interpreted with caution given that the populations and underlying diseases differed markedly across studies. One limitation of the safety analysis is that the AEs and SAEs were not analysed separately.

Some published studies advocate the use of higher doses of teicoplanin, three times per week in an OPAT setting. However, this dose has not been included in this NMA as it is off-label. Furthermore, none of the RCTs included in the NMA used this dosing schedule, hence the concept of equivalence has not been achieved with respect to this dosing schedule for teicoplanin. The use of dalbavancin, vancomycin and linezolid in the clinical studies reflects their intended use in clinical practice. Treatment was administered at the licensed frequency and dose. Thus, the efficacy, safety profile and tolerability expected for dalbavancin in clinical practice are the same as those observed in the clinical program of this antibiotic.

The use of vancomycin and linezolid as the comparator in the dalbavancin studies is not considered a limitation as they are considered part of the current standard of care. However, given the wider range of treatment options available, an indirect treatment comparison was conducted to assess the relative efficacy and safety of dalbavancin versus current standard of care with daptomycin, teicoplanin and tigecycline.

The NMA is based on a systematic literature review [35, 36] which was informed by extensive searches conducted in a range of databases, as well as searches of the websites of key regulatory bodies and relevant conference proceedings, to ensure that as many relevant studies as possible were identified. No limits were placed on date or language. In addition, unpublished data in the form of FDA reports were used to confirm and expand the data set for the NMA. However, the impact of potential publication bias was not explored in the review.

Flaws in the design, conduct and analysis of RCTs could have resulted in bias and raise questions about the validity of the findings. A similarity assessment of studies eligible for inclusion in the NMA was undertaken, as well as a full assessment of risk of bias for each trial identified. The included trials varied in design and quality. A pragmatic approach was taken for the analysis, by including all trials in the NMA regardless of their risk of bias. Whilst some of the criteria assessed (e.g. blinding or methods for handling missing data) may have produced biased results, strict criteria could lead to the exclusion of most trials from the network.

The suitability of trials for inclusion in the NMA was determined by considering whether the studies were suitably homogeneous across the following elements: quality of trials and methods of randomisation; confounding factors in relation to participant populations; confounding factors in relation to circumstances; similarity of treatment arms of interest; and similarity of outcomes of interest. However, there was no discrimination between superiority and non-inferiority trials and whether trials reported meeting their primary endpoint or not.

The assessment of efficacy in the NMA is limited to the ITT populations. Efficacy conclusions regarding antimicrobials are commonly based on the per-protocol, clinically and microbiologically evaluable populations. One of the required assumptions to infer causality is that compared groups can be exchangeable. This assumption does not hold in per-protocol populations. Confounding is almost guaranteed to exist when comparing trial subpopulations, but it is not possible to detect its presence from the available data, let alone to adjust for it.

A particular limitation of the NMA was that each of the comparators involved few trials, with some comparators informed by only one study. However, meta-analyses often include a small number of studies. Furthermore, most of the active treatments are compared directly to vancomycin but not to each other. Hence, comparisons amongst active treatments with no direct information will be subject to more uncertainty than comparisons between vancomycin and the other active treatments. The network has limited power to detect small differences between all the other active treatments. Another limitation is that vancomycin’s efficacy and side-effect profile are often dependent on its serum levels. However, serum levels were not documented in the published vancomycin studies. The completed PRISMA checklist can be found in S4 Table.

## Conclusion

ABSSSI have evolved over a relatively short period of time to become one of the most challenging medical problems in clinical practice. The management of ABSSSI (in particular those due to MRSA) impacts hugely on healthcare systems in terms of the cost of care and consumption of healthcare resources [62]. Hospital stakeholders are constantly searching for new strategies that can improve the quality of care while simultaneously reducing overall expenditure [63]. Dalbavancin affords a promising, new alternative IV antimicrobial agent which has been shown to be as effective as traditional therapies, but with the added benefit of enabling clinicians to treat patients with ABSSSI in a different organisational setting (including the OPAT option). Hence, use of dalbavancin can potentially free-up hospital resources and thereby reduce the costs of hospital admissions and drug administration burden. Notwithstanding, any introduction of an effective treatment with a differential mode of administration into healthcare systems must be followed by a change in clinical practice and patient management in order to fully achieve desirable economic outcomes.

## Supporting information

S1 Table Search strategy for Cochrane library. (DOCX)

S2 Table Search strategy for PubMed. (DOCX)

S3 Table Excluded clinical studies. (DOCX)

S4 Table PRISMA 2009 checklist. (DOC)

## Abbreviations

ABSSSIs: Acute bacterial skin and skin structure infections
AE: Adverse event
cSSSI: Complicated skin and skin structure infections
cSSTI: Complicated skin and soft tissue infection
DAL: Dalbavancin
DAP: Daptomycin
DIC: Deviance Information Criterion
EOT: End of treatment
FDA: Food and Drug Administration
FE: Fixed effect
ITT: Intention to treat
IV: Intravenous
LZD: Linezolid
MRSA: Methicillin-resistant Staphylococcus *Aureus*
MSSA: Methicillin-susceptible Staphylococcus *Aureus*
NMA: Network meta-analysis
OPAT: Outpatient parenteral antibiotic therapy
pD: Leverage or number of parameters
PICOS: Patient, intervention, comparator, outcome, and study design
OR: Odds ratio
RE: Random effect
RCT: Randomised controlled trial
SAE: Serious adverse event
SSSI: Skin and skin structure infections
TIG: Tigecycline
TEI: Teicoplanin
TOC: Test-of-cure visit
VAN: Vancomycin
VRE: Vancomycin-resistant enterococci
