# Supplementary material for: Comparative efficacy and safety of antibiotics used to treat acute bacterial skin and skin structure infections: Results of a network meta-analysis
Source: PLoS One. 2017 Nov 14;12(11):e0187792. doi: 10.1371/journal.pone.0187792 (PMC5685605; doi:10.1371/journal.pone.0187792)
Supplement: S1 Table — (DOCX) [file pone.0187792.s001.docx]

Supplementary Table 1. Search strategy for Cochrane library.

| **ID** | **Search** | **Hits** |
| --- | --- | --- |
| #1 | "ABSSSI":ti,ab,kw or "CSSSI":ti,ab,kw or "SSTI":ti,ab,kw or "CSSTI":ti,ab,kw (Word variations have been searched) | 64 |
| #2 | "acute":ti,ab,kw or "complicated":ti,ab,kw (Word variations have been searched) | 69238 |
| #3 | "SSSI":ti,ab,kw or "BSSSI":ti,ab,kw or "SSTI" (Word variations have been searched) | 29 |
| #4 | "bacterial":ti,ab,kw (Word variations have been searched) | 19784 |
| #5 | "skin structure infection":ti,ab,kw or "skin structure infections":ti,ab,kw or "skin and soft tissue infection":ti,ab,kw or "skin and soft tissue infections":ti,ab,kw (Word variations have been searched) | 394 |
| #6 | #4 AND #5 | 232 |
| #7 | #2 AND (#3 OR #6) | 125 |
| #8 | #1 OR #7 | 149 |
| #9 | MeSH descriptor: [Cellulitis] explode all trees | 116 |
| #10 | MeSH descriptor: [Erysipelas] explode all trees | 18 |
| #11 | MeSH descriptor: [Furunculosis] explode all trees | 9 |
| #12 | MeSH descriptor: [Abscess] explode all trees | 484 |
| #13 | MeSH descriptor: [Wound Infection] explode all trees | 3196 |
| #14 | MeSH descriptor: [Fasciitis] explode all trees | 136 |
| #15 | "cellulitis":ti,ab,kw or "erysipelas":ti,ab,kw or "wound infection":ti,ab,kw or "impetiginous lesion":ti,ab,kw or "furuncle":ti,ab,kw (Word variations have been searched) | 4979 |
| #16 | "furuncles":ti,ab,kw or "infected ulcer":ti,ab,kw or "infected ulcers":ti,ab,kw or "burn":ti,ab,kw or "burns":ti,ab,kw (Word variations have been searched) | 3924 |
| #17 | fasciitis:ti,ab,kw | 262 |
| #18 | "Staphylococcus aureus":ti,ab,kw or "S aureus":ti,ab,kw or "methicillin-susceptible":ti,ab,kw or "methicillin-resistant":ti,ab,kw | 2045 |
| #19 | "MRSA":ti,ab,kw or "Streptococcus pyogenes":ti,ab,kw or "S pyogenes":ti,ab,kw or "Streptococcus agalactiae":ti,ab,kw or "S agalactiae":ti,ab,kw | 864 |
| #20 | "Streptococcus anginosus":ti,ab,kw or "S anginosus":ti,ab,kw or "streptococcus milleri":ti,ab,kw or "S milleri":ti,ab,kw or "Streptococcus intermedius":ti,ab,kw | 14 |
| #21 | "S intermedius":ti,ab,kw or "S constellatus":ti,ab,kw or "streptococcus constellatus":ti,ab,kw | 4 |
| #22 | MeSH descriptor: [Staphylococcal Skin Infections] explode all trees | 164 |
| #23 | #9 OR #10 OR #11 OR #12 OR #13 OR #14 OR #15 OR #16 OR #17 | 9389 |
| #24 | #18 OR #19 OR #20 OR #21 OR #22 | 2551 |
| #25 | #23 AND #24 | 355 |
| #26 | #8 OR #25 | 484 |
