# Supplementary material for: Comparative efficacy and safety of antibiotics used to treat acute bacterial skin and skin structure infections: Results of a network meta-analysis
Source: PLoS One. 2017 Nov 14;12(11):e0187792. doi: 10.1371/journal.pone.0187792 (PMC5685605; doi:10.1371/journal.pone.0187792)
Supplement: S2 Table — (DOCX) [file pone.0187792.s002.docx]

Supplementary Table 2. Search strategy for PubMed.

| **ID** | **Search** | **Hits** |
| --- | --- | --- |
| #1 | "absssi"[Title/Abstract] OR "csssi"[Title/Abstract] OR "ssti"[Title/Abstract] OR "cssti"[Title/Abstract] | 745 |
| #2 | ("acute"[Title/Abstract] OR "complicated"[Title/Abstract]) AND ("sssi"[Title/Abstract] OR "bsssi"[Title/Abstract] OR "ssti"[Title/Abstract]) | 58 |
| #3 | #1 OR #2 | 756 |
| #4 | ("skin structure infection"[Title/Abstract] OR "skin structure infections"[Title/Abstract] OR "skin and soft tissue infection"[Title/Abstract] OR "skin and soft tissue infections"[Title/Abstract]) | 2692 |
| #5 | "cellulitis"[MeSH Terms] | 6661 |
| #6 | "erysipelas"[MeSH Terms] | 1168 |
| #7 | "furunculosis"[MeSH Terms] | 1339 |
| #8 | "abscess"[MeSH Terms] | 48922 |
| #9 | "wound infection"[MeSH Terms] | 37399 |
| #10 | "fasciitis"[MeSH Terms] | 4784 |
| #11 | cellulitis[Title/Abstract] OR erysipelas[Title/Abstract] OR "wound infection"[Title/Abstract] OR "impetiginous lesion"[Title/Abstract] OR "furuncle"[Title/Abstract] OR "furuncles"[Title/Abstract] OR "infected ulcer"[Title/Abstract] OR "infected ulcers"[Title/Abstract] OR "burn"[Title/Abstract] OR "burns"[Title/Abstract] OR "fasciitis"[Title/Abstract] | 70903 |
| #12 | #4 OR #5 OR #6 OR #7 OR #8 OR #9 OR #10 OR #11 | 155148 |
| #13 | "bacterial infections"[MeSH Major Topic] | 609183 |
| #14 | "bacterial"[Title/Abstract] | 313474 |
| #15 | "staphylococcal skin infections"[MeSH Terms] | 4425 |
| #16 | "Staphylococcus aureus"[Title/Abstract] OR "S aureus"[Title/Abstract] OR "methicillin-susceptible"[Title/Abstract] OR "methicillin-resistant"[Title/Abstract] OR MRSA[Title/Abstract] OR "Streptococcus pyogenes"[Title/Abstract] OR "S pyogenes"[Title/Abstract] OR "Streptococcus agalactiae"[Title/Abstract] OR "S agalactiae"[Title/Abstract] OR "Streptococcus anginosus"[Title/Abstract] OR "S anginosus"[Title/Abstract] OR "streptococcus milleri" OR "S milleri"[Title/Abstract] OR "Streptococcus intermedius"[Title/Abstract] OR "S intermedius"[Title/Abstract] OR "S constellatus"[Title/Abstract] OR "streptococcus constellatus"[Title/Abstract] | 86857 |
| #17 | #13 OR #14 OR #15 OR #16 | 912180 |
| #18 | #12 AND #17 | 39229 |
| #19 | #3 OR #18 | 39597 |
| #20 | Dalbavancin[Title/Abstract] OR "171500-79-1" | 201 |
| #21 | Linezolid[Title/Abstract] OR "165800-03-3" | 3744 |
| #22 | "Vancomycin"[MeSH Terms] OR Vancomycin[Title/Abstract] OR "1404-90-6" | 21179 |
| #23 | "clindamycin"[MeSH Terms] OR "Clindamycin"[Title/Abstract] OR "18323-44-9" | 9576 |
| #24 | "Daptomycin"[MeSH Terms] OR "Daptomycin"[Title/Abstract] OR "103060-53-3" | 1993 |
| #25 | "Ceftaroline"[Title/Abstract] OR "400827-46-5" | 197691 |
| #26 | "doxycycline"[MeSH Terms] OR "Doxycycline"[Title/Abstract] OR "564-25-0" | 12346 |
| #27 | "trimethoprim sulfamethoxazole combination"[MeSH Terms] OR "trimethoprim sulfamethoxazole"[Title/Abstract] OR " 738-70-5" | 13817 |
| #28 | Tigecycline[Title/Abstract] OR "220620-09-7" | 1683 |
| #29 | Oritavancin[Title/Abstract] OR "171099-57-3" | 186557 |
| #30 | #20 OR #21 OR #22 OR #23 OR #24 OR #25 OR #26 OR #27 OR #28 OR #29 | 428385 |
| #31 | (“double blind”[Title/Abstract] OR “double blinded"[Title/Abstract] OR “double-blind”[Title/Abstract] OR “double-blinded”[Title/Abstract] OR RCT[Title/Abstract] OR Randomised[Title/Abstract] OR randomised[Title/Abstract] OR controlled[Title/Abstract] OR controlled[Title/Abstract] OR control[Title/Abstract] OR Placebo[Title/Abstract] OR Trial[Title/Abstract]) | 2503128 |
| #32 | (Study[Title/Abstract] OR studies[Title/Abstract]) AND (open[Title/Abstract] OR open-label[Title/Abstract] OR non-randomised[Title/Abstract] OR non-randomized[Title/Abstract] OR observational[Title/Abstract] OR "case series"[Title/Abstract]) | 244470 |
| #33 | #31 OR #32 | 2676706 |
| #34 | #19 AND #30 AND #33 | 657 |
| #35 | ("animals"[MeSH Terms]) NOT humans[MeSH Terms] | 3949251 |
| #36 | #34 NOT #35 | 606 |
