# Supplementary material for: Comparative efficacy and safety of antibiotics used to treat acute bacterial skin and skin structure infections: Results of a network meta-analysis
Source: PLoS One. 2017 Nov 14;12(11):e0187792. doi: 10.1371/journal.pone.0187792 (PMC5685605; doi:10.1371/journal.pone.0187792)
Supplement: S3 Table — (DOCX) [file pone.0187792.s003.docx]

Supplementary Table 3. Excluded Clinical Studies.

| **Reference** | **Reason for exclusion** |
| --- | --- |
| Bochud-Gabellon I, Regamey C. Teicoplanin, a new antibiotic effective against gram-positive bacterial infections of the skin and soft tissues. Dermatologica. 1988;176(1):29-38. | Retrospective observational study |
| Cenizal MJ, Skiest D, Luber S, Bedimo R, Davis P, Fox P, Delaney K, Hardy RD. Prospective randomized trial of empiric therapy with trimethoprim-sulfamethoxazole or doxycycline for outpatient skin and soft tissue infections in an area of high prevalence of methicillin-resistant Staphylococcus aureus. Antimicrob Agents Chemother. 2007l;51(7):2628-30. | Lack of reported data/definitions/outcomes |
| Chen AF, Wessel CB, Rao N. Staphylococcus aureus screening and decolonization in orthopaedic surgery and reduction of surgical site infections. Clin Orthop Relat Res. 2013l;471(7):2383-99. | Review article |
| Chen YS, Lee SC, Kim WJ. Efficacy and tolerability of linezolid in treating severe skin and soft tissue infections caused by Gram-positive pathogens. J Formos Med Assoc. 2004;103(5):349-54. | Phase 4, single arm, open label study |
| Chuang YC, Chang CM, Aradhya S, Nagari B, Pai V, Dartois N, Jouve S, Cooper A Efficacy and safety of tigecycline monotherapy compared with vancomycin-aztreonam in the treatment of complicated skin and skin structure infections in patients from India and Taiwan. Journal of Microbiology, Immunology, and Infection] 2011; 44(2): 116-124 | Pooled analysis of [44] and [47] |
| Davis SL, McKinnon PS, Hall LM, Delgado G Jr, Rose W, Wilson RF, Rybak MJ. Daptomycin versus vancomycin for complicated skin and skin structure infections: clinical and economic outcomes. Pharmacotherapy. 2007;27(12):1611-8. | Observational study |
| Davis SL, Rybak MJ, Amjad M, Kaatz GW, McKinnon PS. Characteristics of patients with healthcare-associated infection due to SCCmec type IV methicillin-resistant Staphylococcus aureus. Infect Control Hosp Epidemiol. 2006;27(10):1025-31. | Results not reported separately for SSTIs |
| Duane TM, Capitano B, Puzniak LA, Biswas P, Joshi M. The impact of linezolid versus vancomycin on surgical interventions for complicated skin and skin structure infections caused by methicillin-resistant Staphylococcus aureus. Surg Infect (Larchmt). 2013;14(4):401-7. | Subgroup analysis of [50] |
| Duane TM, Weigelt JA, Puzniak LA, Huang DB Linezolid and vancomycin in treatment of lower-extremity complicated skin and skin structure infections caused by methicillin-resistant Staphylococcus aureus in patients with and without vascular disease Surgical infections. 2012; 13(3): 147-53 | Pooled analysis of [15] and [50] |
| Eagye KJ, Kim A, Laohavaleeson S, Kuti JL, Nicolau DP. Surgical site infections: does inadequate antibiotic therapy affect patient outcomes? Surg Infect (Larchmt). 2009;10(4):323-31. | No interventions of interest |
| Eckmann C, Dryden M. Treatment of complicated skin and soft-tissue infections caused by resistant bacteria: value of linezolid, tigecycline, daptomycin and vancomycin. Eur J Med Res. 2010;15(12):554-63. | Review article |
| Ellis-Grosse EJ, Babinchak T, Dartois N, Rose G, Loh E; Tigecycline 300 cSSSI Study Group; Tigecycline 305 cSSSI Study Group. The efficacy and safety of tigecycline in the treatment of skin and skin-structure infections: results of 2 double-blind phase 3 comparison studies with vancomycin-aztreonam. Clin Infect Dis. 2005;41 Suppl 5:S341-53. | Pooled analysis of [44] and [47] |
| Evers R, Antony NI, Alozie O, Antony S Pilot study comparing daptomycin and telavancin in the treatment of skin and soft tissue infections. Internet Journal of Infectious Diseases. 2013; 12 (2):1-4. | Study of uncomplicated SSTIs |

| Garau J, Ostermann H, Medina J, Avila M, McBride K, Blasi F; REACH study group. Current management of patients hospitalized with complicated skin and soft tissue infections across Europe (2010-2011): assessment of clinical practice patterns and real-life effectiveness of antibiotics from the REACH study. Clin Microbiol Infect. 2013;19(9):E377-85. | No interventions of interest |
| --- | --- |
| Gardiner D, Dukart G, Cooper A, Babinchak T. Safety and efficacy of intravenous tigecycline in subjects with secondary bacteremia: pooled results from 8 phase III clinical trials. Clin Infect Dis. 2010;50(2):229-38. | Pooled analysis of [44] and [47] |
| Konychev A, Heep M, Moritz RKC, Kreuter A, Shulutko A, Fierlbeck G, Bouylout K, Pathan R, Chaves R A comparative randomised clinical trial against semisynthetic penicillins and glycopeptides supports the use of daptomycin as first-line treatment of complicated skin and soft-tissue infections in the elderly. Clinical Microbiology and Infection. Conference: 22nd European Congress of Clinical Microbiology and Infection | Already reported in [54] |
| Lassus A. Comparative studies of azithromycin in skin and soft-tissue infections and sexually transmitted infections by Neisseria and Chlamydia species. J Antimicrob Chemother. 1990;25 Suppl A:115-21. | No interventions of interest |
| Lipsky BA, Stoutenburgh U. Daptomycin for treating infected diabetic foot ulcers: evidence from a randomized, controlled trial comparing daptomycin with vancomycin or semi-synthetic penicillins for complicated skin and skin-structure infections. J Antimicrob Chemother. 2005;55(2):240-5. | Pooled analysis of [45] |
| Pallin DJ, Binder WD, Allen MB, Lederman M, Parmar S, Filbin MR, Hooper DC, Camargo Jr CA Clinical trial: Comparative effectiveness of cephalexin plus trimethoprim-sulfamethoxazole versus cephalexin alone for treatment of uncomplicated cellulitis: A randomized controlled trial. Clinical infectious diseases. 2013; 56(12): 1754-62 | Study of uncomplicated SSSIs |
| Peppard WJ, Daniels A, Fehrenbacher L, Winner J. Evidence based approach to the treatment of community-associated methicillin-resistant Staphylococcus aureus. Infect Drug Resist. 2009;2:27-40. | Review article |
| Pertel PE, Eisenstein BI, Link AS, Donfrid B, Biermann EJ, Bernardo P, Martone WJ The efficacy and safety of daptomycin vs. vancomycin for the treatment of cellulitis and erysipelas. International Journal of Clinical Practice. 2009; 63(3): 368-75. | Study of uncomplicated SSSIs |
| Schmitz GR, Bruner D, Pitotti R, Olderog C, Livengood T, Williams J, Huebner K, Lightfoot J, Ritz B, Bates C, Schmitz M, Mete M, Deye G. Randomized controlled trial of trimethoprim-sulfamethoxazole for uncomplicated skin abscesses in patients at risk for community-associated methicillin-resistant Staphylococcus aureus infection. Ann Emerg Med. 2010;56(3):283-7. | Study does not report on ABSSSIs |
| Shen HN, Lu CL. Skin and soft tissue infections in hospitalized and critically ill patients: a nationwide population-based study. BMC Infect Dis. 2010;10:151. | No interventions of interest |
| Stromberg BV, Reines HD, Hunt P. Comparative clinical study of Sulbactam and ampicillin and clindamycin and tobramycin in infections of soft tissues. Surg Gynecol Obstet. 1986;162(6):575-8. | No interventions of interest |
| Van Laethem Y, Hermans P, De Wit S, Goosens H, Clumeck N. Teicoplanin compared with vancomycin in methicillin-resistant Staphylococcus aureus infections: preliminary results. J Antimicrob Chemother. 1988;21 Suppl A:81-7. | Study does not report on ABSSSIs |

| Vasilev K, Reshedko G, Orasan R, Sanchez M, Teras J, Babinchak T, Dukart G, Cooper A, Dartois N, Gandjini H, Orrico R, Ellis-Grosse E; 309 Study Group. A Phase 3, open-label, non-comparative study of tigecycline in the treatment of patients with selected serious infections due to resistant Gram-negative organisms including Enterobacter species, Acinetobacter baumannii and Klebsiella pneumoniae. J Antimicrob Chemother. 2008;62 Suppl 1:i29-40. | Non-randomized study |
| --- | --- |
| Weigelt J, Kaafarani HM, Itani KM, Swanson RN. Linezolid eradicates MRSA better than vancomycin from surgical-site infections. Am J Surg. 2004;188(6):760-6. | Subset of surgical-site-infection patients in [15] |
| Weigelt JA, Lipsky BA, Tabak YP, Derby KG, Kim M, Gupta V. Surgical site infections: Causative pathogens and associated outcomes. Am J Infect Control. 2010;38(2):112-20. | No interventions of interest |
| Wilcox MH. Efficacy of tigecycline in complicated skin and skin structure infections and complicated intra-abdominal infections. J Chemother. 2005;17, Suppl 1:23-9. | Review article |
